# Genomic characterization and virulence gene profiling of *Erysipelothrix rhusiopathiae* isolated from widespread muskox mortalities in the Canadian Arctic Archipelago

**DOI:** 10.1186/s12864-024-10592-9

**Published:** 2024-07-14

**Authors:** Lakshmi Vineesha Seru, Taya L. Forde, Amélie Roberto-Charron, Fabien Mavrot, Yan D. Niu, Susan J. Kutz

**Affiliations:** 1 Faculty of Veterinary Medicine, University of Calgary, Calgary, AB Canada; 2 School of Biodiversity, One Health & Veterinary Medicine, University of Glasgow, Glasgow, UK; 3 Government of Nunavut, Department of Environment, Iqaluit, NU Canada

**Keywords:** Arctic, *Ovibos moschatus*, Epidemic, *Erysipelothrix rhusiopathiae*, Virulence genes, Pathogenicity islands, Prophages, Pan-genome wide association studies

## Abstract

**Background:**

Muskoxen are important ecosystem components and provide food, economic opportunities, and cultural well-being for Indigenous communities in the Canadian Arctic. Between 2010 and 2021, *Erysipelothrix rhusiopathiae* was isolated from carcasses of muskoxen, caribou, a seal, and an Arctic fox during multiple large scale mortality events in the Canadian Arctic Archipelago. A single strain (‘Arctic clone’) of *E. rhusiopathiae* was associated with the mortalities on Banks, Victoria and Prince Patrick Islands, Northwest Territories and Nunavut, Canada (2010–2017). The objectives of this study were to (i) characterize the genomes of *E. rhusiopathiae* isolates obtained from more recent muskox mortalities in the Canadian Arctic in 2019 and 2021; (ii) identify and compare common virulence traits associated with the core genome and mobile genetic elements (i.e. pathogenicity islands and prophages) among Arctic clone versus other *E. rhusiopathiae* genomes; and iii) use pan-genome wide association studies (GWAS) to determine unique genetic contents of the Arctic clone that may encode virulence traits and that could be used for diagnostic purposes.

**Results:**

Phylogenetic analyses revealed that the newly sequenced *E. rhusiopathiae* isolates from Ellesmere Island, Nunavut (2021) also belong to the Arctic clone. Of 17 virulence genes analysed among 28 Arctic clone isolates, four genes – adhesin, rhusiopathiae surface protein-A (*rsp*A), choline binding protein-B (*cbp*B) and CDP-glycerol glycerophosphotransferase (*tag*F) – had amino acid sequence variants unique to this clone when compared to 31 other *E. rhusiopathiae* genomes. These genes encode proteins that facilitate *E. rhusiopathiae* to attach to the host endothelial cells and form biofilms. GWAS analyses using Scoary found several unique genes to be overrepresented in the Arctic clone.

**Conclusions:**

The Arctic clone of *E. rhusiopathiae* was associated with multiple muskox mortalities spanning over a decade and multiple Arctic islands with distances over 1000 km, highlighting the extent of its spatiotemporal spread. This clone possesses unique gene content, as well as amino acid variants in multiple virulence genes that are distinct from the other closely related *E. rhusiopathiae* isolates. This study establishes an essential foundation on which to investigate whether these differences are correlated with the apparent virulence of this specific clone through in vitro and in vivo studies.

**Supplementary Information:**

The online version contains supplementary material available at 10.1186/s12864-024-10592-9.

## Background

**Fig. 1:**
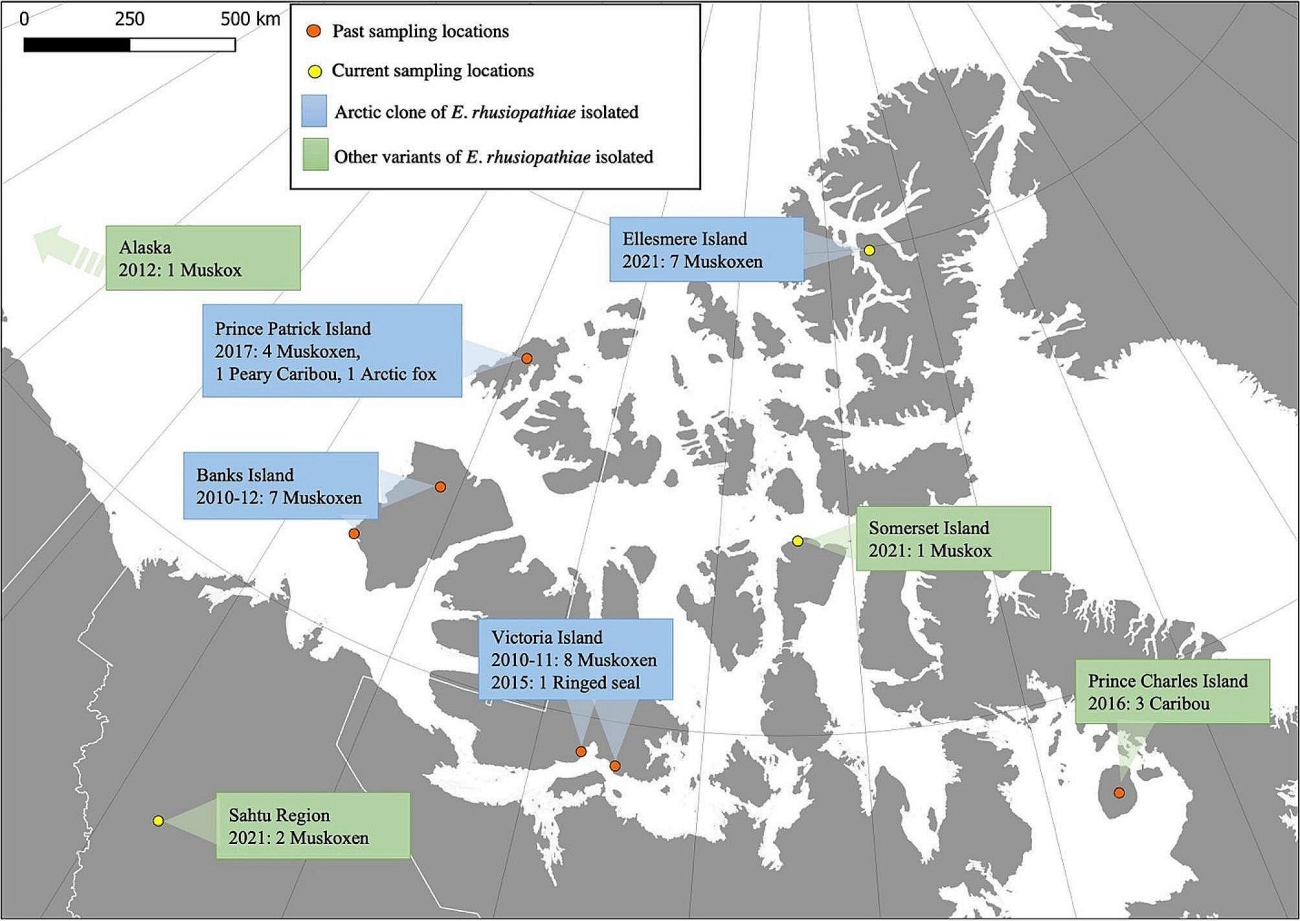
Map summarizing the origin of *Erysipelothrix rhusiopathiae* isolated from dead Arctic wildlife and included in genomic characterization and virulence gene profiling. Locations where the Arctic clone of *E. rhusiopathiae* has been identified are indicated in blue. All locations with the Arctic clone were linked with acute mortality events in muskoxen

Muskoxen (*Ovibos moschatus*) are large-bodied herbivores that are well adapted to the Arctic environment. They provide food, economic opportunities, and cultural well-being for Indigenous people in the Canadian Arctic [1]. Accelerated Arctic warming and emerging infectious diseases [2–5], together with a limited gene pool [6] and other stressors are increasingly threatening the future of this species [7–9]. Since 2010, the bacterium *Erysipelothrix rhusiopathiae* has emerged as a significant cause of mortality in muskoxen in the Canadian Arctic [10]. *E. rhusiopathiae* is a Gram-positive, opportunistic, facultative intracellular bacterium best known from domestic livestock, where it is generally transmitted via contaminated feed and water [11]. This bacterium is most commonly associated with domestic pigs [12, 13] in which three clinical manifestations are described: a severe acute septicaemic form, a milder subacute urticarial form and a chronic form presenting with arthritis and/or endocarditis [14]. Muskoxen have presented with the severe acute septicaemic form, where animals in good body condition appear to have died rapidly [10]. In wildlife more broadly, *E. rhusiopathiae* is associated with sporadic individual mortalities and occasional disease outbreaks have been reported in birds [15], rodents [16], and marine mammals [17, 18]. Although *E. rhusiopathiae* has been circulating in Canadian muskox populations since at least the 1970s [19], it was first identified as a disease-causing agent in 2010. Between 2010 and 2013 a single clonal lineage, the ‘Arctic clone’, part of the phylogenetic clade 3 of *E. rhusiopathiae* [20], was cultured from muskox carcasses during mass mortality events on Banks and Victoria Islands, Northwest Territories and Nunavut, Canada [10, 21] (Fig. 1). In 2015, this same strain was cultured from a dead seal on Victoria Island, and in summer 2017, it was cultured from carcasses of muskoxen (*n* = 4), Peary caribou (*Rangifer tarandus pearyi*) (*n* = 6) and an Arctic fox (*Vulpes lagopus*) (*n* = 1) on Prince Patrick Island, Northwest Territories (NWT), Canada [22]. *E. rhusiopathiae* was isolated from other sporadic mortalities of muskoxen, caribou and moose in temperate, sub- and mid Arctic Canada and Alaska during this same timeframe; however, whole genome sequencing of the isolates from these sources revealed various genotypes, representative of the wide population diversity of this species [21, 22]. *E. rhusiopathiae* has been isolated from more recent muskox mortality events in the eastern Canadian Arctic, Nunavut (NU) and western Canadian mainland, Northwest Territories (NWT) and are sequenced and characterized as part of the present study.

Muskox populations affected by the Arctic clone of *E. rhusiopathiae* experienced substantial population declines during and following the outbreaks [7, 22, 23]. Understanding the drivers of the emergence and apparently high morbidity and mortality rates associated with the Arctic clone is one of the first steps in developing mitigation and management strategies. Considering the epidemiological triad, plausible explanatory factors for the observed mortalities associated with this pathogen include increased host susceptibility due to genetic bottlenecks [6, 24] or increasing stressors [25]; changing environmental factors facilitating bacterial survival and transmission [26, 27]; or increased pathogen virulence. Based on the unusually high mortality and rapidity of deaths in muskoxen infected with the Arctic clone, we hypothesize that this genotype contains unique virulence traits compared to other closely related isolates.

Virulence factors of bacteria influence their pathogenicity, such as the ability to evade the host immune system, leading to infection and potentially disease progression. Virulence factors may be encoded by genes in the chromosomal DNA or by plasmids, and are often carried by different mobile genetic elements such as pathogenicity islands (PAIs), prophages, or transposons [28]. *E. rhusiopathiae* is a facultative intracellular pathogen that replicates in phagocytic cells, and its pathogenicity is related to cellular attachment, and intracellular invasion and survival [29, 30]. Previously identified PAIs and prophages in *E. rhusiopathiae* have been shown to harbour transposases, as well as several genes responsible for antimicrobial resistance [31, 32], and could thus play a role in the horizontal transfer of virulence genes.

Although virulence factors of porcine *E. rhusiopathiae* are generally well characterized, data on those of *E. rhusiopathiae* isolated from wild animals are lacking. Initial screening for 59 putative virulence genes among *E. rhusiopathiae* genomes isolated from widespread geographical locations, including the Arctic clone, in 2016 [21], demonstrated that the majority of these genes were found to be present in most genomes (i.e., are core genes). At that time, only gene presence/absence was investigated. However, changes in the DNA sequences encoding these genes could lead to alterations in the amino acid sequences, potentially changing protein structure and function [33]. To understand the unique pathogenic potential associated with the Arctic clone, identifying variations in its virulence genes is important.

The aim of this study was to genomically characterize *E. rhusiopathiae* isolated from widespread mortalities of muskoxen and other Arctic wildlife, with a focus on genomic differences that might be related to virulence. Our specific objectives were to: (1) determine whether recently recovered *E. rhusiopathiae* isolates from eastern NU archipelago and western NWT mainland belong to the Arctic clone; (2) compare sequences of virulence genes and assess for specific mobile genetic elements (PAIs and prophages) of interest among Arctic clone and non-Arctic clone isolates; and (3) determine if there is genetic content unique to the Arctic clone.

## Methods

### Sample collection and processing

Ethical approval for sample collection was obtained from the University of Calgary Animal Care Committee (Protocol number AC18-0093). Wildlife Research Permits were obtained from the Governments of Nunavut (WL2016-058, WL2021-053, WL2019-051, WL2021-048) and the Northwest Territories (WL 500,664). A total of 57 samples from three geographic locations were screened for the presence of *E. rhusiopathiae* (Fig. 1). (1) Fifty-two samples were collected opportunistically during the investigation of an unusual mortality event of muskoxen on the Fosheim Peninsula, Ellesmere Island, NU, Canada in August 2021. Collections comprised 29 fecal and tissue samples from 10 muskox carcasses (including lung, liver, kidney, heart, spleen, bone marrow, intestine), as well as 22 fecal samples from two apparently healthy muskox herds in the area. (2) Bone marrow samples were collected from the skeletal remains of two dead muskoxen found on Somerset Island, NU, Canada in July 2019 and 2021. (3) One bone marrow sample was collected from a recently dead adult muskox in August 2021, and three tissue samples (lung, liver, kidney) from a dead muskox calf found in September 2021 from the Sahtu region, NWT, Canada. All samples were frozen at -20 ℃ upon collection and shipped within three months from the collection sites. Sample processing and analyses were performed at the Faculty of Veterinary Medicine, University of Calgary, Alberta, Canada.

### Isolation and identification of *E. rhusiopathiae*

For recovery of *E. rhusiopathiae*, samples were thawed at room temperature (est. 22 ℃). One-gram subsamples of tissue or fecal samples were mechanically homogenized using gentleMACS™ Octo Dissociator (Miltenyi Biotec, Inc., San Diego, CA, USA) in 10 ml brain heart infusion (BHI) broth (BD, Sparks, MD, USA) supplemented with 5% horse serum and incubated for 24 − 48 hours (h) at 37 ℃ with 5% CO_2_. Subsequently, 300 µl of enriched culture was further incubated for 48 h in 2.7 ml modified BHI (mBHI) supplemented with 5% horse serum, kanamycin (40 µg/ml) (VWR International, Mississauga, Ontario, Canada), neomycin (50 µg/ml) (VWR International) and vancomycin (25 µg/ml) (VWR International), as previously described for the selective culture of this bacterium [20, 34]. To assess the colony morphology, culture positive samples were sub-cultured to selective media containing sodium azide (MilliporeSigma, Oakville, Ontario, Canada) and crystal violet agar (Ward’s Science, Rochester, New York, USA) (SACV) and incubated for 48 h at 37 ℃ with 5% CO_2_. A single colony from SACV agar that showed very small, transparent, and circular colonies was sub-streaked on 5% sheep blood agar (TSA with sheep blood) (ThermoScientific, USA) to obtain pure colonies for DNA extraction [35]. For molecular confirmation of species identity, one loopful of purified colonies from blood agar was suspended in 200 µl sterile phosphate buffer saline (PBS) for DNA extraction using DNeasy blood and tissue kit (Qiagen, Mississauga, ON, Canada) following manufacturer’s instructions. The confirmation of isolates was performed by qPCR using primers and a species-specific probe targeting the 3’ non-coding region of the rRNA gene cluster as described by Pal et al. [36]. *E. tonsillarum* was simultaneously ruled out (in multiplex) by including a second species-specific probe described in the same publication.

### Library preparation, sequencing, and *de novo* assembly

Whole genome sequencing of *E. rhusiopathiae* isolates from Ellesmere Island, Somerset Island and the Sahtu region was outsourced to the Centre de diagnostic vétérinaire de l’Université de Montréal, Saint Hyacinthe, Quebec, Canada. At their facility, DNA extracted from isolates was quantified using Qubit™ dsDNA HS Assay Kit and a Qubit™ fluorometer (Thermofisher Scientific, MA, USA). Sequencing libraries were prepared with Nextera XT DNA Library Preparation Kit (Illumina, CA, USA) following standard protocols. Library quality was assessed using Agilent High Sensitivity DNA Kit in a Bioanalyzer (Agilent, CA, USA). Libraries were sequenced on a v3 600-cycle cartridge using a MiSeq instrument with PhiX spiked in at 1% (Illumina, CA, USA). The reads obtained were assessed for quality using FASTQC v.0.11.9 in Galaxy and the reads were trimmed for quality and adaptors using Trimmomatic v.0.36 [37] within the CLIMB computing platform for microbial genomics [38]. The reads of low-quality were removed using a sliding window (4:20) and MINLEN (50) parameters. The *de novo* assembly of newly-sequenced genomes was performed with SPAdes tool v.3.15.4 [39] also within CLIMB. Final quality assessment of scaffolds was performed using QUAST v.4.6.3 [40] (i.e., to determine the number of contigs, GC%, assembly length, and N50). Assemblies thus obtained were further assessed using Kmerfinder tool v.3.2 to confirm the species assignation [41]. The good quality assemblies with length (~ 1.8 mb) and GC content (~ 36.6%) consistent with the genome of *E. rhusiopathiae* and no evidence of contamination based on Kmerfinder were used for further analyses (*n* = 9); one exception was made for a tenth genome (593-EU04-K), where 2% of the assembly was classified as *Pseudomonas aeruginosa*. In addition, 45 high quality (coverage > 30X) whole genome sequences of previously characterized *E. rhusiopathiae* isolates, along with reference strains Fujisawa (GenBank accession no. AP012027), SY1027 (GenBank accession no. CP005079), WH13013 (GenBank accession no. CP017116), and ATCC19414 (GenBank accession no. NZ_ACLK00000000) were included in the core-genome alignment (Supplementary Table S1, Additional File 1). The dataset comprised *E. rhusiopathiae* isolates from a variety of hosts, including from muskoxen, caribou, moose, seal, swine, poultry, and marine mammals as previously described [20, 21]. Most of the genomes included were from Clade 3, although representation was included from other clades for use as an outgroup.

### Core genome alignment

A core genome alignment, consisting of the subset of genetic regions conserved across the 57 *E. rhusiopathiae de novo* assemblies (clade 1 genomes excluded), was carried out using the Parsnp tool within the Harvest suite [42] through CLIMB. Parsnp identifies the orthologous sequences conserved in all the aligned genomes, from which it estimates the phylogenetic relationship using a maximum likelihood approach. The resulting phylogeny was visualized and edited with iTOL v6 [43].

### Identification of amino acid variability among *E. rhusiopathiae* virulence genes

A total of 17 putative virulence genes were selected based on previous experimental studies and those that have been linked with certain pathogenic mechanisms of *E. rhusiopathiae* (Table 1). Nucleotide sequences of virulence genes from *E. rhusiopathiae* strain Fujisawa available on GenBank were downloaded and used to create custom BLAST databases in Geneious v.10.2. [44]. We searched for the presence of these 17 virulence genes in all *de novo* assemblies (*n* = 59) by BLASTn searches, where a BLAST hit of 95% pairwise identity was considered positive for a particular virulence gene. Nucleotide sequences were extracted from the positive hits and translations to amino acid sequences were performed within Geneious using translation Table 1 for bacteria as previously described [45]. Amino acid rather than nucleotide sequences were compared in order to focus on differences with a higher likelihood of phenotypic/functional relevance. Multiple sequence alignment to identify the amino acid variability was performed using CLUSTAL-2.1 implemented within Geneious, and variants were recorded in comparison with Fujisawa reference sequence. Phylogenetic trees for each gene were estimated from the multiple sequence alignment using a Neighbor-Joining method implemented in Geneious Tree Builder, using the Jukes-Cantor model.

**Table 1:** Virulence genes of *Erysipelothrix rhusiopathiae* commonly reported in the literature that were assessed for among genomes in this study. Nucleic acid sequences were translated to amino acid (AA) sequences

Locus tag	Name/Description	Position*	AA length & no. of variations detected	Variants specific to the Arctic clone**	References
EL194_RS00590	MetQ/NIpA family ABC transporter substrate binding protein	129,726–130,514	262 (10)	No	[46]
ERH_0094	Surface protective antigen (*spaA*)	112,931–114,811	626 (37)	No	[29, 47]
ERH_0432	CDP-glycerol glycerophosphotransferase (*tagF*)	471,205–472,350	381 (16)	Yes	[48]
ERH_0728	Leucine rich repeat protein	765,909–767,441	510 (135)	No	[49]
ERH_1472	Internalin like protein	1,555,822–1,557,471	549 (21)	No	[49]
ERH_1356	Adhesin	1,418,103–1,419,023	306 (15)	Yes	[29]
ERH_0768	Choline binding protein-B (*cbpB*)	813,020–814,840	606 (24)	Yes	[50]
ERH_0668	Rhusiopathiae surface protein-A (*rspA*); biofilm formation, protective antigen	701,546–707,524	1992 (73)	Yes	[51, 52]
ERH_0669	Rhusiopathiae surface protein-B (*rspB*); biofilm formation	707,761–710,133	808 (38)	No	[31, 51]
ERH-0150	Hyaluronidase (*hylA*)	175,894–178,998	1034 (42)	No	[29, 31]
ERH_0299	Neuraminidase (*nanH*)	331,513–335,109	1198 (64)	No	[29, 53]
ERH_0072	Patatin like phospholipase	74,708–75,616	302 (3)	No	[29]
ERH_0388	Phospholipase D	417,904–418,650	248 (9)	No	[29]
ERH_0333	Cardiolipin synthetase (*cls*)	363,488–365,014	508 (5)	No	[29]
ERH_0334	Patatin like phospholipase B	365,022–365,876	284(6)	No	[29]
ERH_1534	Glycerol-3-phosphate dehydrogenase (GAPDH)	1,618,401–1,619,405	334 (0)	No	[54]
ERH_1433	Lysophospholipase C	1,512,331–1,513,185	284 (10)	No	[29]

*Positions are in relationship with reference strain Fujisawa (AP012027)

** Amino acid variants present in all or nearly all Arctic clone isolates and absent in most other isolates investigated

### Genome annotation and identification of PAIs and prophages

*De novo* assemblies were annotated using Prokka v.1.13 [55] within CLIMB. The resulting Genbank files were used as input into IslandViewer-4 software [56]. Prophages were searched for among the genomes using PHASTER tool (Phage Search Tool- Enhanced Release) [57]. This was done by uploading *de novo* assemblies in FASTA format to the online version of this tool (https://phaster.ca/). All tools were implemented using default settings.

### Identification of unique gene content using Pan-GWAS analysis

A pan-genome wide association study (GWAS) approach was taken to identify gene content unique to the Arctic clone in comparison with other *E. rhusiopathiae* genomes. The Prokka-annotated genomes in general feature format (.gff) were used to analyse the pan-genome using Roary, using default settings [58]. The gene presence/absence file generated by Roary, along with a trait file, were used to establish gene clusters associated with the Arctic clone using Scoary [59]. The trait file was created in Microsoft excel by classifying all the isolates into binary categories, i.e., Arctic clone isolates were indicated as “1” and others as “0”. The *p* values for multiple testing and managing error-I were adjusted using the Benjamin-Hochberg method within Scoary. This method orders original *p*-values from smallest to largest and then the hypotheses are accepted or rejected to minimize the detection of false positives [60]. The list of significant genes for the Arctic clone generated by Scoary comprised both characterized (annotated) and hypothetical genes. We searched for gene function of annotated proteins using UniProt database [61].

### Data availability

All newly sequenced genomes in this work were submitted to European Nucleotide Archive under project accession number PRJEB73478.

## Results

### Culture of *E. rhusiopathiae* from dead muskoxen from Fosheim Peninsula, Ellesmere Island, and other Arctic locations

Of the 35 samples cultured for *E. rhusiopathiae* from 14 muskox carcasses, 30 (85.7%) were culture positive (Supplementary Table S2, Additional File 1). Samples from one carcass each from Ellesmere Island and Somerset Island did not culture positive. Samples that showed growth were confirmed as *E. rhusiopathiae* using qPCR. In general, dead muskoxen were in good body condition. Several had bleeding from the nose and anus, and/or had serosanguinous fluid in the thorax and peritoneal cavities. None of the 22 fecal samples collected from two herds of apparently healthy muskoxen on Ellesmere Island cultured positive for *E. rhusiopathiae*.

### Phylogenetic relationships of newly isolated *E. rhusiopathiae* compared with previously characterized isolates

Ten whole genome sequences newly-generated in this study were included in our genomic analyses. i) Nine whole genome sequences of *E. rhusiopathiae* isolated from Ellesmere (6) and Somerset (1) Islands and the Sahtu region (2) passed quality thresholds and were used for downstream analyses (Supplementary Table S3, Additional File 1). The contigs had an average N50 of 106,167 base pairs and an average of 104 contigs per assembly. ii) One additional genome of 593-EU04-K, isolated from an Ellesmere Island muskox, which had a lower N50 (79,868) and a higher number of contigs (257), was also included to assess at least one isolate per culture-positive muskox carcass.

**Fig. 2:**
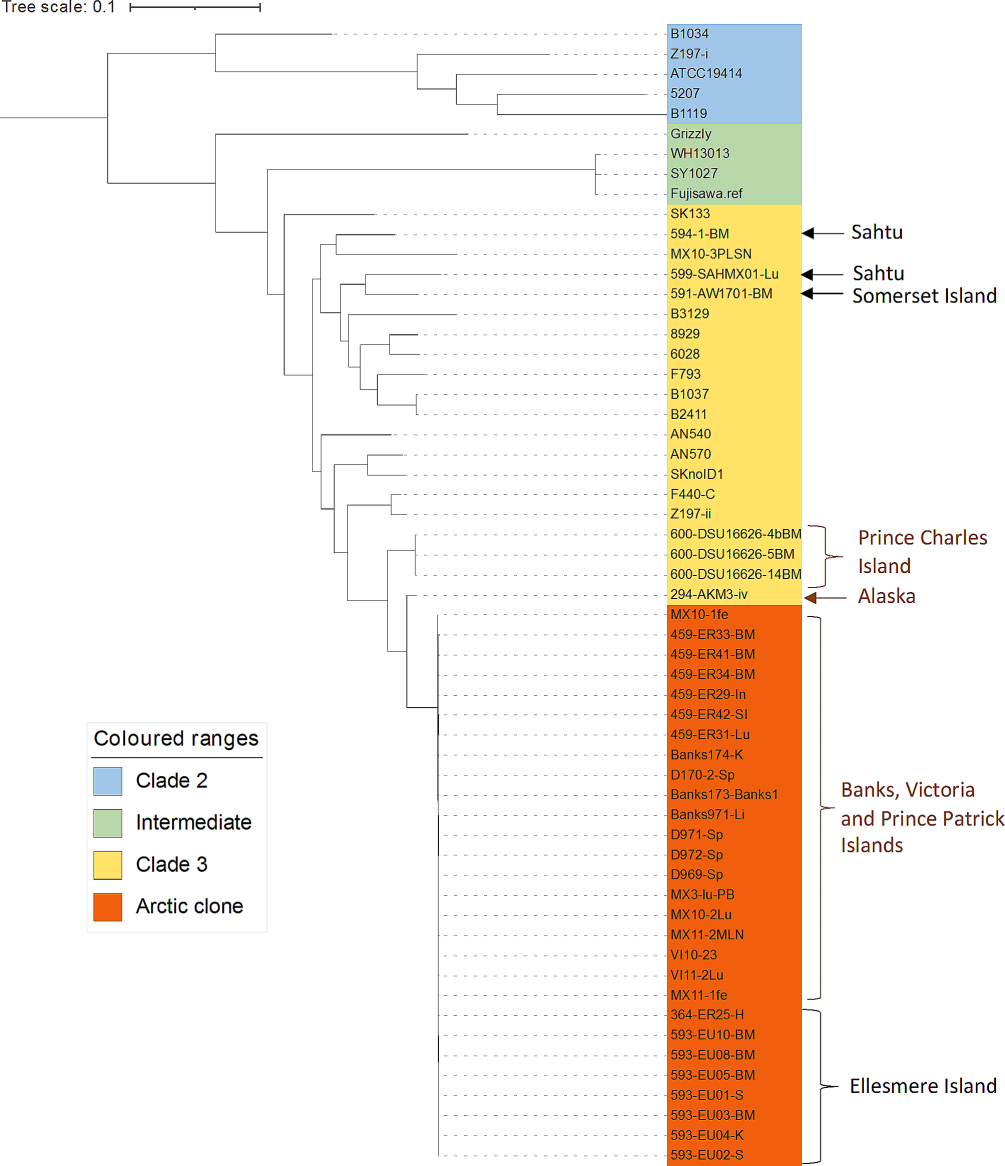
Maximum likelihood tree of *Erysipelothrix rhusiopathiae* whole genome sequences estimated using Parsnp [42]. Tree is rooted to clade 2 genomes. Sequences generated as part of this study are shown in black, whereas previously sequenced Arctic genomes are indicated in brown. Isolate details are provided in Supplementary Table S1, Additional File 1

The phylogenetic tree comprising 57 *E. rhusiopathiae* genomes, including 43 previously-characterised genomes, showed that *E. rhusiopathiae* isolates from Ellesmere Island fell within clade 3, and clustered together with *E. rhusiopathiae* from Banks, Victoria, and Prince Patrick Islands; these were isolated from muskox die-offs with symptoms of acute death and previously identified as being part of the Arctic clone (Fig. 2). Conversely, isolates from the Sahtu region (594-1-BM and 599-SAHMX01-Lu) and Somerset Island (591-AW1701-BM) muskoxen, which clustered within clade 3, were genetically distinct from the Arctic clone and from each other. Isolate AKM3-iv, from a muskox in Alaska [21], remained the closest isolate to the Arctic clone, with the next closest isolates from Prince Charles Island caribou, where mortalities were assumed to be caused by a severe weather-related starvation event [62, 63].

### Amino acid variance between the Arctic clone and other closely related isolates

**Fig. 3:**
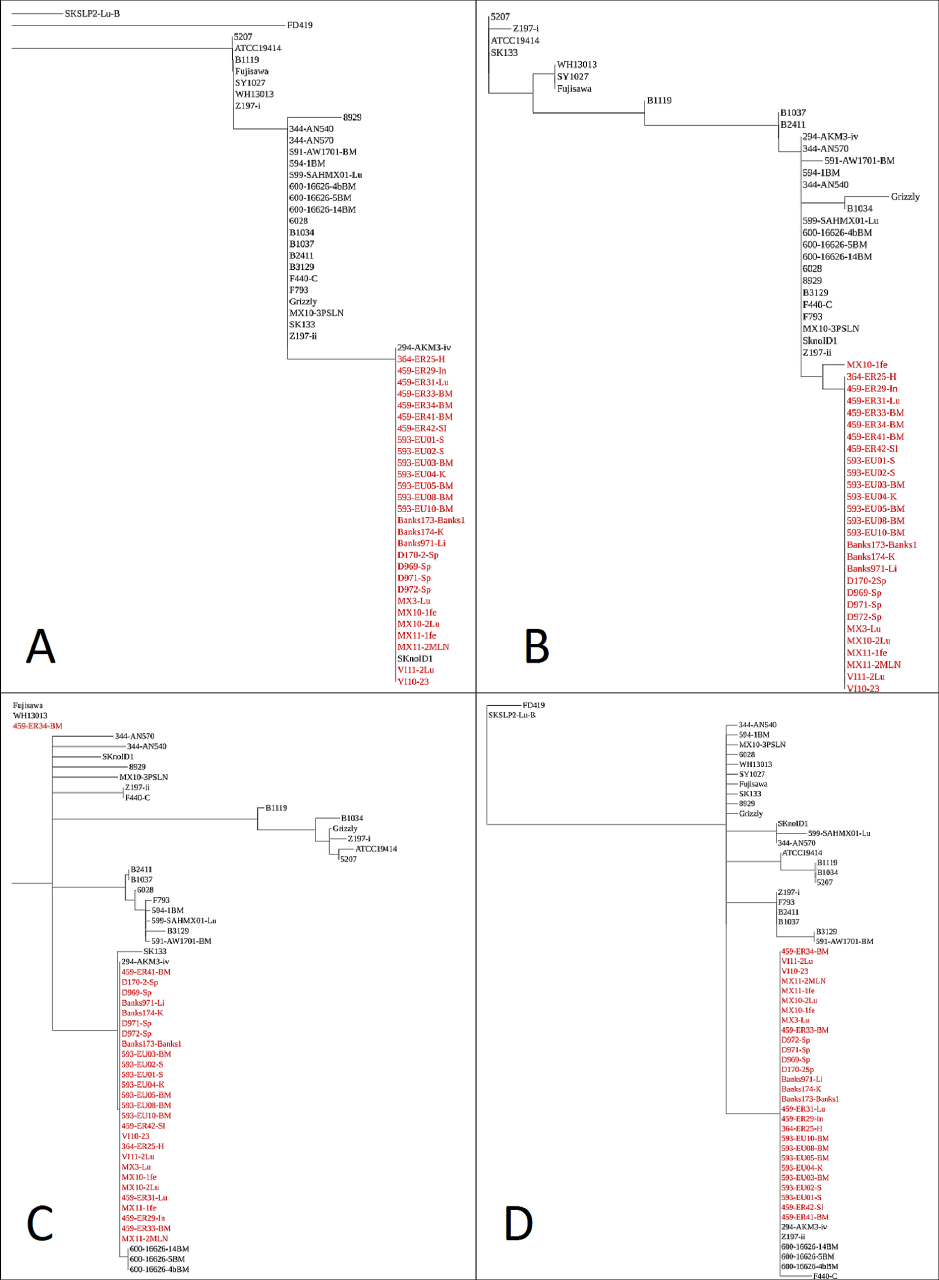
Virulence genes with distinct amino acid sequence patterns in the Arctic clone of *Erysipelothrix rhusiopathiae* compared with other genomes. Gene trees were estimated using the Neighbor-Joining method implemented in Geneious Tree Builder [44]. Genomes belonging to the Arctic clone are shown in red. **(A)** adhesin; **(B)** choline binding protein-B; **(C)** rhusiopathiae surface protein-A; and **(D)** CDP-glycerol glycerophosphotransferase

Of the 17 virulence genes investigated, 16 were present in all genomes, four of which were highly conserved, i.e., with a maximum of 9 AA variations among all 59 genomes: GAPDH, patatin like phospholipase B, patatin like phospholipase, and phospholipase D. Internalin was present in only 17/59 *E. rhusiopathiae* isolates examined. Four genes had specific amino acid variants that were present in all or nearly all Arctic clone isolates and absent in most of the other isolates investigated. These were adhesin, choline binding protein-B (*cbpB*), rhusiopathiae surface protein-A (*rspA*), and CDP-glycerol glycerophosphotransferase (*tagF*) (Fig. 3). Adhesin had 15 amino acid variations overall, two of which (V97I and D122G) were unique to the Arctic clone and two other clade 3 isolates (SKnoID1 (British Columbia, CA) and AKM3-iv (Alaska, US). Similarly, choline binding protein-B had 24 variations, two of which (R95L and N495D) were unique to the Arctic clone except genome MX10-1fe (Victoria Island, NWT, CA), which had the N495D variant but not R95L. Rhusiopathiae surface protein-A showed a higher number of variations, with variants at 73 (3.7%) of 1992 amino acid positions. With the exception of isolate 459-ER34-BM (Prince Patrick Island, NWT, CA), the Arctic clone sequences for this protein clustered separately, along with that of AKM3-iv (Alaska, US). Within CDP-glycerol glycerophosphotransferase sequences, a single amino acid variation at position 223 (D223N) was unique to the Arctic clone and 6 other isolates [AKM3-iv (Alaska, US), 600-16626-4bBM, 600-16266-5BM, 600-16626-14BM (Prince Charles Island, NU, CA), F440-C (Alberta, CA) and Z197-ii (Alberta, CA)]. However, F440-C had an additional variation at the position 104 (R104Q) which led to this isolate segregating from others in the tree (Fig. 3D).

Only *hylA* and *spaA* had insertions variably present among the genomes. For instance, the Arctic clone and a limited number of non-Arctic clone isolates exhibited the presence of a tandem repeat in the *hylA* gene consisting of four amino acids (ETKP) repeated four times starting from position 984, whereas other isolates had fewer than four copies of the repeat motif (Supplementary Fig. S1, Additional File 1).

### Identification of pathogenicity islands and prophages

We screened for the presence of virulence genes within PAIs using the IslandViewer-4 tool (Supplementary Table S4, Additional File 1). Over 183 PAIs of variable length and location were predicted across all 59 *E. rhusiopathiae* genomes investigated. In general, genomes isolated from swine showed a higher number of PAIs, whereas the Arctic clone isolates each had 2–3 PAIs. All of the PAIs harboured genes associated with horizontal transfer such as transposase, integrase, recombinase, and prophage genes. PAIs from porcine isolates (B3129, 6028, 8929) harboured genes encoding antibiotic resistance such as aminoglycoside 6-adenylyltransferase, streptomycin 3’-adenylyltransferase, tetracycline resistance (*tetM)* from transposon TnFO1, bleomycin resistance protein, and kanamycin nucleotidyltransferase. Isolates other than those of swine origin in clade 3 contained putative virulence genes identified as collagen adhesin, CFA/I fimbrial subunit D, multidrug export protein *MepA*, transcriptional regulatory protein *SrrA*, and Internalin-J. Finally, two putative toxin-encoding genes were found within PAI of several genomes, including within the Arctic clone: NAD (+)—arginine ADP- ribosyl transferase *EFV*, and toxin B.

The PHASTER online tool [57] identified prophages present in all 59 *E. rhusiopathiae* genomes. A total of 137 prophages accounting for 3 intact, 126 incomplete and 8 questionable prophages were found. Incomplete and questionable prophages were present in most of the *E. rhusiopathiae* genomes and ranged from 5.9 to 62.4 kb in length. All the Arctic clone genomes carried 2–3 prophages each. Meanwhile, non-Arctic clone genomes carried between 1 and 4 prophages. A specific prophage 11.2 kb long was found in the Arctic clone, as well as in one other non-Arctic clone isolate (594-1BM). However, none of the prophages harboured known virulence genes.

### Identification of gene content unique to the Arctic clone through pan-GWAS

Scoary identified a total of 101 genes that were significantly (*p* < 0.05) associated with the Arctic clone (Supplementary Table S5, Additional File 2). Of those, 69 genes were annotated as hypothetical proteins with unknown function. The top 15 annotated genes with highest sensitivity and specificity are shown in Table 2.

**Table 2:** Annotated genes most significantly associated with the Arctic clone of *Erysipelothrix rhusiopathiae*. Predicted through pan-GWAS using Scoary

Gene	Annotation	Sensitivity^a^	Specificity^b^	*p* value^c^
*hhaIM_1*	DNA methyltransferase	100	100	1.81E-17
*xerD_1*	Tyrosine recombinase	100	87.09	8.93E-13
*espL*	Putative sugar transferase	100	77.42	1.23E-10
*tarL*	Ribitol-5-phosphate cytidylyltransferase 2	100	77.42	1.23E-10
*arnB*	UDP-4-amino-4-deoxy-L-arabinoseoxoglutarate	100	77.42	1.23E-10
*tarF*	Teichoic acid poly(glycerol phosphate) polymerase	100	77.42	1.23E-10
*group_1458*	Beta-N-acetylhexosaminidase	100	70.96	2.29E-09
*toxB*	Toxin B	100	67.74	8.77E-09
*nahK_2*	N-acetylhexosamine 1-kinase	100	64.52	3.14E-08
*noc_1*	Nucleoid occlusion protein	100	58.06	3.39E-07
*cpsY*	Exopolysaccharide phosphotransferase	100	58.06	3.39E-07
*group_1036*	Putative ABC transporter permease	100	58.06	3.39E-07
*lexA*	Levansucrase	100	58.06	3.39E-07
*radD*	Putative DNA repair helicase	100	51.61	3.02E-06
*group_140*	NAD(+)—arginine ADP- ribosyltransferase	100	51.61	3.02E-06

^a^Sensitivity is calculated as the proportion of the Arctic clone genomes that have the gene, whilst ^b^specificity is the proportion of the non-Arctic clone genomes that do not have the gene. ^c^The *p* values for multiple testing and managing error I were calculated using the Benjamin Hochberg method within Scoary [59].

### Predicted functions of unique genes found in the Arctic clone

Based on analyses using Uniprot [61], the top hit genes from Scoary had predicted functions related to DNA binding and repair. More specifically, four genes are involved in DNA binding: *hhalM_1* encodes a DNA methyltransferase enzyme that is a component of restriction modification systems [64]; *xerD* encodes a phage integrase SAM-like protein that has role in DNA binding [65]; *noc_1* encodes a nucleoid occlusion protein; *lexA* encodes a DNA-binding helix turn helix protein responsible for DNA binding and serine-type endopeptidase activity. The DNA repair gene *radD* is involved in alteration of one or more nucleotide sites in DNA through a DNA repair helicase protein. The N-acetylhexosamine 1-kinase encoded by *nahK_2* regulates phosphorylation responsible for cleaving DNA and integrating phage-related fragments. The gene *cpsY* encodes an exopolysaccharide phosphotransferase enzyme that has a role in exopolysaccharide synthesis. *Group_1036* encodes a putative ABC transporter permease enzyme. Two genes are involved in teichoic acid biosynthesis process: the gene *tarF* encodes teichoic acid poly (glycerol phosphate) polymerase and *tarL* gene encodes Ribitol-5-phosphate cytidylyltransferase 2. The gene *arnB* encodes a UDP-4-amino-4-deoxy-L-arabinoseoxoglutarate that helps in intracellular protein transport. Finally, two toxin-associated genes were among the top Scoary hits with predicted functions: *Group_140* encodes NAD (+)—arginine ADP- ribosyltransferase *EFV* (Uniprot ID Q838U8), and *toxB* gene encodes toxin B (Uniprot ID P18177).

## Discussion

### New understanding of the geographical distribution of the Arctic clone

Phylogenetic analysis of *E. rhusiopathiae* isolates from the Fosheim Peninsula, Ellesmere Island, analyzed alongside a global collection of genomes, confirmed that the Arctic clone is present in this geographical location. The disease presentation was similar to what had been previously described in other locations, i.e. summer mortalities, animals of all age and sex classes and in good condition, septicemia and acute death, and bleeding from natural orifices [10]. Ellesmere Island is located over 1000 km to the northeast of Banks and Victoria Islands, where the Arctic clone was first identified in association with muskox mortalities starting in 2010. Wildlife health surveillance in the Arctic is extremely limited due to vast distances and sparse human populations. It is thus not clear how long the Arctic clone has been present on the Fosheim peninsula, how widespread it is, and what its impacts are on muskoxen or other species. Further research looking into its distribution in the eastern Arctic Archipelago, environmental persistence, host range and transmission, as well as establishing a molecular clock to understand patterns of spread, are underway. The finding of diverse strains of *E. rhusiopathiae* on Somerset Island and in the Sahtu region is consistent with previous observations that a wide variety of strains of this bacterium are found in wildlife in other locations [21, 22], and emphasizes the novelty of finding this unique clone across such a wide geographic area.

### Virulence gene sequence variants unique to the Arctic clone

The variations we observed in the amino acid sequences – as predicted from the whole genome sequence data – have the potential to alter protein production and activity (e.g., through changes in shape or residual charge) [66], and is thus one of the potential mechanisms that could alter the virulence of particular strains. We found that the Arctic clone exhibited unique amino acid sequences in 4 out of the 17 virulence genes investigated in this study. Of these four genes, two (adhesin, and choline binding protein B) are related to adhesion of *E. rhusiopathiae* to host endothelial cells [49, 50]. In addition, rhusiopathiae surface protein A is involved in adherence to abiotic and biotic surfaces through biofilm formation [52]. For pathogens to withstand host immunological mechanisms and to deploy available virulence genes, adhesion is a universal prerequisite [67]. Furthermore, CDP-glycerol glycerophosphotransferase has been suggested to encode a component of the cell wall, contributing to the structural integrity and pathogenic potential of the bacterium [48].

In addition to these unique virulence gene variants, we identified different copy numbers of a 4-amino acid tandem repeat motif in the hyaluronidase (*hylA*) gene in the Arctic clone, as well as some non-Arctic clone isolates, whereas other genomes showed 2–3 copies. Variability in tandem repeat units of different genes has been previously associated with virulence and stress-related strategies amongst strains of *Salmonella* spp. [68]. Hyaluronidase is an important virulence factor produced by bacteria, helminths and arthropods, and is hypothesized to be a spreading factor that disseminates these pathogens into tissues [69]. Although some studies have suggested a role of *hylA* in *E. rhusiopathiae* pathogenesis [70], its importance in virulence has been heavily disputed for this species [69, 71]. In addition to the unknown relevance of these differential repeats, there is also some uncertainty in the *de novo* assembly of repetitive regions of DNA using short sequence reads, such as those generated using Illumina. These limitations include potential assembly errors, incomplete assemblies, and challenges in accurately resolving complex repeat structures due to short read lengths, which complicate the understanding and analysis of these genomic regions [72]. The addition of long-read data, e.g., using Nanopore, would increase the confidence in the assembly of such genomic regions. Overall, further studies to assess potential functional implications of these predicted unique amino acid differences in the Arctic clone could include using structural predictions, as well as in vitro and in vivo studies.

While the Arctic clone genomes harbored 2–3 prophages, none of the genes they carried had putative roles in virulence. These mobile elements therefore seem less likely to be linked to any unique functional differences of this strain. Some of the PAI detected carried potential virulence genes that Scoary detected as being over-represented in the Arctic clone compared with other genomes, as discussed below.

### Genes associated with the Arctic clone

Scoary analyses found several unique genes to be overrepresented in the Arctic clone compared to other closely related genomes. A high proportion of genes (69/101 = 68%) were hypothetical, i.e., with unknown function. Of the top 15 genes associated with the Arctic clone having a predicted function, it is perhaps not surprising that most are involved in DNA recombination and repair. *E. rhusiopathiae* is highly recombinogenic [20] and these genes are necessary for integration of foreign genetic material [73]. Further supporting the importance of recombination in this bacterium, several of these unique genes were among those found on pathogenicity islands, including tyrosine recombinase *XerD*, nucleoid occlusion protein *Noc_1*, and putative DNA repair helicase *RadD*. Moreover, of note was the detection of two genes annotated as putative toxins – toxin B and NAD (+)—arginine ADP- ribosyltransferase *EFV* – carried on PAIs of *E. rhusiopathiae*. No toxin has ever been associated with this bacterium previously [11]. The gene annotated as toxin B by Prokka was 1827 nucleotides in length. While the Uniprot reference (P18177) was to an exotoxin produced by *Clostridium difficile* that causes cytotoxicity (7692 bp) [74], there was low homology between the sequences. This gene has been previously annotated as N-acetylmuramoyl-L-alanine amidase family protein in other *E. rhusiopathiae* genomes (e.g., RNM30934.1). The gene annotated as NAD (+)—arginine ADP- ribosyl transferase *EFV* by Prokka was 1581 nucleotides in length. The Uniprot reference for this predicted protein (Q838U8) is associated with *Enterococcus faecalis* (1461 bp) [75]. This gene has been previously annotated as a minor capsid protein of *E. rhusiopathiae* (e.g., WP_115354732.1), and is likely a component of a bacteriophage. BLAST searches of both genes did not find any significant homology in other bacterial species. Investigations into the function of these two genes, particularly whether they act as toxins, would be of high interest. One annotated gene, *hhaIM_1*, showed perfect sensitivity and specificity, and thus represents a good candidate for the development of a PCR assay for the rapid confirmation of the Arctic clone; this is currently under development by our team.

## Conclusion

*E. rhusiopathiae* has emerged as an important and enigmatic pathogen of muskoxen and other Arctic species. It is associated with widespread mortality events, high mortality rates and concomitant to population declines of muskoxen in the Canadian Arctic archipelago and other populations [10, 23]. Our work demonstrates that a single, apparently highly pathogenic strain, the Arctic clone, first detected in the western Arctic Archipelago in 2010, is now present on the Fosheim Peninsula of Ellesmere Island, to the far northeast. This Arctic clone possesses several unique genes, including putative toxin-encoding genes, and distinct amino acid variations in several conserved virulence genes, all of which may be playing a role in the apparent high pathogenicity of this strain. Understanding the functional significance of these differences, together with unraveling the factors related to the pathogen, a genetically depauperate host [6], and a rapidly changing environment [76] in the observed outbreaks, are critical for understanding the impacts of this pathogen on muskoxen, other species, and the broader Arctic ecosystem. The comprehensive comparative genome analysis presented here will guide further studies (e.g., in vitro or challenge studies) to elucidate the genetic and functional basis of the pathogenesis of *E. rhusiopathiae*.

### Electronic supplementary material

Below is the link to the electronic supplementary material.

Supplementary Material 1. Additional File 1, Table S1. *Erysipelothrix rhusiopathiae* genomes included in this study. Table S2. Culture results for *Erysipelothrix rhusiopathiae* from tissue samples collected from muskox mortalities. Table S3. Quality metrics of 10 newly sequenced *E. rhusiopathiae* genome assemblies, as determined by QUAST. Table S4. Prophages and pathogenicity islands (PAIs) detected in 59 *Erysipelothrix rhusiopathiae* genomes. Fig. S1. Multiple sequence alignment of hyaluronidase amino acid sequence from 57 *E. rhusiopathiae* genomes.

Supplementary Material 2. Additional File 2. Table S5. Scoary results for the significant genes associated with the Arctic clone.

## Data Availability

Sequence data generated for this manuscript are available at European Nucleotide Archive under project accession number PRJEB73478. Other publicly available sequences included in this study are described in Supplementary Table S1, Additional File 1.
