# Supplementary material for: Genomic characterization and virulence gene profiling of Erysipelothrix rhusiopathiae isolated from widespread muskox mortalities in the Canadian Arctic Archipelago
Source: BMC Genomics. 2024 Jul 14;25:691. doi: 10.1186/s12864-024-10592-9 (PMC11247837; doi:10.1186/s12864-024-10592-9)
Supplement: Supplementary file 1 — Supplementary Material 1. Additional File 1, Table S1. Erysipelothrix rhusiopathiae genomes included in this study. Table S2. Culture results for Erysipelothrix rhusiopathiae from tissue samples collected from muskox mortalities. Table S3. Quality metrics of 10 newly sequenced E. rhusiopathiae genome assemblies, as determined by QUAST. Table S4. Prophages and pathogenicity islands (PAIs) detected in 59 Erysipelothrix rhusiopathiae genomes. Fig. S1. Multiple sequence alignment of hyaluronidase amino acid sequence from 57 E. rhusiopathiae genomes. [file 12864_2024_10592_MOESM1_ESM.docx]

# **Supplementary Information for:**

# **Genomic Characterization and Virulence Gene Profiling of *Erysipelothrix rhusiopathiae* Isolated from Widespread Wildlife Mortalities in the Canadian Arctic Archipelago**

Lakshmi Vineesha Seru, Taya L. Forde, Amélie Roberto-Charron, Fabien Mavrot, Yan D. Niu, Susan J. Kutz

**Table S1. *Erysipelothrix rhusiopathiae* genomes included in this study.**

| **Animal ID (New sample accession)** | **Clade** | **Species** | **Year Sampled** | **Location** | **Sample Type** | **Reference** |
| --- | --- | --- | --- | --- | --- | --- |
| 593-EU01-S* (ERS18601147) | 3 | Muskox | 2021 | Ellesmere Island, NU, Canada | Spleen | This study^#^ |
| 593-EU02-S* (ERS18601148) | 3 | Muskox | 2021 | Ellesmere Island, NU, Canada | Spleen |  |
| 593-EU03-BM* (ERS18601149) | 3 | Muskox | 2021 | Ellesmere Island, NU, Canada | Bone marrow |  |
| 593-EU04-K* (ERS18601150) | 3 | Muskox | 2021 | Ellesmere Island, NU, Canada | Kidney |  |
| 593-EU05-BM* (ERS18601151) | 3 | Muskox | 2021 | Ellesmere Island, NU, Canada | Bone marrow |  |
| 593-EU08-BM* (ERS18601152) | 3 | Muskox | 2021 | Ellesmere Island, NU, Canada | Bone marrow |  |
| 593-EU10-BM* (ERS18601153) | 3 | Muskox | 2021 | Ellesmere Island, NU, Canada | Bone marrow |  |
| 591-AW1701-BM (ERS18601146) | 3 | Muskox | 2019 | Somerset Island, NU, Canada | Bone marrow |  |
| 594-1BM (ERS18601155) | 3 | Muskox | 2021 | Norman Wells, Sahtu, NWT, Canada | Bone marrow |  |
| 599-SAHMX01-Lu (ERS18601154) | 3 | Muskox | 2021 | Sahtu, NWT, Canada | Lung |  |
| WH13013 | Int | Swine | 2013 | China | Spleen | [1] |
| Fujisawa | Int | Swine | Pre 1985 | Japan |  | [2] |
| SY1027 | Int | Swine | Unknown | China | Blood | [3] |
| ATCC19414 | 2 | Swine | Pre 1987 | Unknown | Spleen | NZ_ACLK00000000.2 |
| SKSLP2-Lu | 1 | Caribou | 2013 | BC, Canada | Lung | [4] |
| FD419 | 1 | Bottlenose dolphin | 1997 | Sea World Australia | Skin |  |
| B1034 | 2 | Poultry | 2006 | Belgium | Unknown |  |
| Z197-i | 2 | Caribou | 2014 | AB, Canada | Bone marrow |  |
| B1119 | 2 | Poultry | 2008 | Belgium | Ovarium |  |
| 5207 | 2 | Bottlenose dolphin | 1990 | Belgium | Unknown |  |
| Grizzly | Int | Caribou | 2014 | AB, Canada | Bone marrow |  |
| SK133 | 3 | Caribou | 2013 | BC, Canada | Bone marrow |  |
| B3129 | 3 | Swine | 2012 | Belgium | Liver |  |
| 6028 | 3 | Swine | 1998 | QC, Canada | Unknown |  |
| 8929 | 3 | Swine | 1996 | QC, Canada | Unknown |  |
| F793 | 3 | Caribou | 2013 | AB, Canada | Bone marrow |  |
| B2411 | 3 | Poultry | 2008 | Belgium | Liver |  |
| B1037 | 3 | Poultry | 2007 | Belgium | Unknown |  |
| MX10-3PSLN | 3 | Muskox | 2010 | Victoria Island, NWT, Canada | Prescapular Lymph node |  |
| AN540 | 3 | Moose | 2014 | BC, Canada | Bone marrow |  |
| SKnoID1 | 3 | Caribou | 2013 | BC, Canada | Bone marrow |  |
| AN570 | 3 | Moose | 2014 | BC, Canada | Bone marrow |  |
| Z197-ii | 3 | Caribou | 2014 | AB, Canada | Bone marrow |  |
| F440-C | 3 | Caribou | 2013 | AB, Canada | Bone marrow |  |
| AKM3-iv | 3 | Muskox | 2012 | Alaska-NSP | Bone marrow |  |
| MX10-1fe* | 3 | Muskox | 2010 | Victoria Island, NWT, Canada | Feces |  |
| MX10-2Lu* | 3 | Muskox | 2010 | Victoria Island, NWT, Canada | Lung |  |
| MX3-Lu* | 3 | Muskox | 2010 | Victoria Island, NWT, Canada | Lung |  |
| MX11-1fe* | 3 | Muskox | 2011 | Victoria Island, NWT, Canada | Feces |  |
| VI11-2Lu* | 3 | Muskox | 2011 | Victoria Island, NWT, Canada | Lung |  |
| VI10-23* | 3 | Muskox | 2010 | Victoria Island, NWT, Canada | Tonsil |  |
| MX11-2MLN* | 3 | Muskox | 2011 | Victoria Island, NWT, Canada | Mesenteric Lymph node |  |
| D969-Sp* | 3 | Muskox | 2012 | Banks Island, NWT, Canada | Spleen |  |
| Banks173-Banks1* | 3 | Muskox | 2012 | Banks Island, NWT, Canada | Kidney |  |
| Banks971-Li* | 3 | Muskox | 2012 | Banks Island, NWT, Canada | Liver |  |
| D972-Sp* | 3 | Muskox | 2012 | Banks Island, NWT, Canada | Spleen |  |
| D971-Sp* | 3 | Muskox | 2012 | Banks Island, NWT, Canada | Spleen |  |
| D170-2-Sp* | 3 | Muskox | 2012 | Bank Island, NWT, Canada | Spleen |  |
| Banks174-K* | 3 | Muskox | 2012 | Banks Island, NWT, Canada | Kidney |  |
| 600-16626-4bBM (ERS18379759) | 3 | Caribou | 2016 | Prince Charles Island, NU, Canada | Bone marrow | [5]^#^ |
| 600-16626-5BM (ERS18379760) | 3 | Caribou | 2016 | Prince Charles Island, NU, Canada | Bone marrow |  |
| 600-16626-14BM (ERS18379761) | 3 | Caribou | 2016 | Prince Charles Island, NU, Canada | Bone marrow |  |
| 364-ER25-H* (ERS18379762) | 3 | Seal | 2015 | Victoria Island, NWT, Canada | Heart |  |
| 459-ER33-BM* (ERS18379763) | 3 | Muskox | 2017 | Prince Patrick Island, NWT, Canada | Bone marrow |  |
| 459-ER34-BM* (ERS18379764) | 3 | Peary Caribou | 2017 | Prince Patrick Island, NWT, Canada | Bone marrow |  |
| 459-ER41-BM* (ERS18379765) | 3 | Fox | 2017 | Prince Patrick Island, NWT, Canada | Bone marrow |  |
| 459-ER31-Lu* (ERS18379766) | 3 | Muskox | 2017 | Prince Patrick Island, NWT, Canada | Lung |  |
| 459-ER42-SI* (ERS18379767) | 3 | Muskox | 2017 | Prince Patrick Island, NWT, Canada | Small Intestine |  |
| 459-ER29-In* (ERS18379768) | 3 | Muskox | 2017 | Prince Patrick Island, NWT, Canada | Intestine |  |

Isolate names marked with (*) belong to the Arctic clone; Int- intermediate clade, BC- British Columbia, AB- Alberta, QC- Quebec, NWT- Northwest Territories, NU- Nunavut

^#^ Sequence data available on ENA under project accession number PRJEB73478.

**Table S2. Culture results for *Erysipelothrix rhusiopathiae* from tissue samples collected from muskox mortalities.** One carcass was sampled on Somerset Island in 2019 (AW1701). All other carcasses on Ellesmere and Somerset Islands and the Sahtu region were sampled in 2021.

| Location | Animal ID | Heart | Lung | Spleen | Liver | Intestine | Bone marrow | Kidney | Feces |
| --- | --- | --- | --- | --- | --- | --- | --- | --- | --- |
| Ellesmere Island | 593-EU01 |  |  | + |  |  | + |  |  |
|  | 593-EU02 | + | + | + | + | + | + | + | + |
|  | 593-EU03 |  |  |  |  |  | + |  |  |
|  | 593-EU04 | + | + | + | + |  | + | + |  |
|  | 593-EU05 |  |  |  |  |  | + |  |  |
|  | 593-EU06 | + |  |  |  |  | - |  |  |
|  | 593-EU07 |  |  |  |  |  | - |  |  |
|  | 593-EU08 |  |  |  |  |  | + |  |  |
|  | 593-EU09 | + | + | + | + |  | + | + |  |
|  | 593-EU10 |  |  |  |  |  | + |  |  |
| Somerset Island | 591-AW1302 |  |  |  |  |  | - |  |  |
|  | 591-AW1701 |  |  |  |  |  | + |  |  |
| Sahtu region | 594-1 |  |  |  |  |  | + |  |  |
|  | 599-SAHMX01 |  | + |  | - |  |  | - |  |

(+) Positive & (-) Negative for *E. rhusiopathiae* using culture and confirmatory qPCR. Blank cells indicate no sample tested.

**Table S3. Quality metrics of 10 newly sequenced *E. rhusiopathiae* genome assemblies, as determined by QUAST.**

| **Genome ID** | **Size (bp)** | **GC (%)** | **No. of contigs** | **N50*** |
| --- | --- | --- | --- | --- |
| 593-EU01-S | 1762771 | 36.41 | 111 | 79408 |
| 593-EU02-S | 1783193 | 36.64 | 211 | 79868 |
| 593-EU03-BM | 1761098 | 36.4 | 76 | 79868 |
| 593-EU04-K | 1814448 | 37.22 | 257 | 79868 |
| 593-EU05-BM | 1758690 | 36.47 | 76 | 95271 |
| 593-EU08-BM | 1757800 | 36.47 | 90 | 79868 |
| 593-EU10-BM | 1755910 | 36.43 | 161 | 71814 |
| 591-AW1701-BM | 1767724 | 36.31 | 92 | 103617 |
| 594-1BM | 1823411 | 36.26 | 65 | 123506 |
| 599-SAHMX01-Lu | 1749086 | 36.35 | 51 | 242281 |

*N50 is the length of the contig that - when all contigs are ordered largest to smallest - when added, covers half of the bases of the assembly.

**Table S4. Prophages and pathogenicity islands (PAIs) detected in 59 *Erysipelothrix rhusiopathiae* genomes.** Prophages were detected using PHASTER and PAIs using IslandViewer-4**.**

| **Animal ID** | **Prophage**  **Region length (kb)** | **Prophage**  **Completeness** | **Specific Keywords** | **Number of PAIs** | **Virulence-associated Genes detected in PAIs** |
| --- | --- | --- | --- | --- | --- |
| SKSLP2-Lu | 11.2 | Incomplete | Protease, Tail | 5 | Endo-alpha-N-acetylgalactosaminidase |
|  | 7.7 | Incomplete | NA |  |  |
|  | 7 | Incomplete | NA |  |  |
|  | 10.5 | Incomplete | Transposase |  |  |
| FD419 | 11.2 | Incomplete | Protease, Tail | 5 | Endo-alpha-N-acetylgalactosaminidase, Internalin-J |
|  | 7 | Incomplete | NA |  |  |
| B1034 | 18.9 | Incomplete | Integrase | 3 | Collagen adhesin,  Transcriptional regulatory protein (SrrA),  NAD(+)—arginine ADP-ribosyltransferase (EFV) |
|  | 20.8 | Questionable | Tail, Terminase, Portal, Head |  |  |
| Z197-i | 25.2 | Incomplete | Integrase | 5 | Internalin-J, Cold shock-like protein (CspLA), SrrA |
|  | 20.3 | Incomplete | Terminase, Portal, Head, Tail |  |  |
| ATCC19414 | 7.1 | Incomplete | Tail, Transposase | 3 | Internalin-J |
| B1119 | 44 | Intact | Integrase, Terminase, Portal, Protease, Capsid, Tail | 4 | Fosfomycin resistance protein (FosX),  Endoribonuclease (ToxN) |
|  | 13.3 | Incomplete | Recombinase, Tail |  |  |
| 5207 | 21.8 | Incomplete | Integrase | 5 | Collagen adhesin, EFV |
|  | 37.1 | Incomplete | Head, Tail, Integrase |  |  |
|  | 18.4 | Incomplete | Terminase, Head, Tail |  |  |
| Fujisawa | 8.4 | Incomplete | Integrase, Capsid | 3 | EFV |
|  | 36.3 | Questionable | Integrase, Terminase, Head, Tail |  |  |
|  | 7.1 | Incomplete | Tail, Transposase |  |  |
|  | 7.8 | Incomplete | Transposase |  |  |
| SY1027 | 10.2 | Questionable | Tail, Transposase | 2 | - |
|  | 9.5 | Incomplete | Transposase, Tail |  |  |
|  | 7.8 | Incomplete | Transposase |  |  |
|  | 29.6 | Incomplete | Integrase, Capsid |  |  |
| 591-AW-17-01-BM | 7 | Incomplete | NA | 4 | - |
| 599-SAH-MX01-Lu | 5.9 | Incomplete | Transposase, Tail | 3 | - |
| WH13013 | 62.4 | Questionable | Tail, Head, Terminase, Integrase | 3 | EFV |
|  | 28.9 | Incomplete | Capsid, Transposase |  |  |
| Grizzly | 34.6 | Intact | Integrase, Tail, Terminase, Portal, Protease, Capsid | 5 | - |
| SK133 | 17.1 | Incomplete | Integrase | 3 | EFV |
|  | 25.7 | Incomplete | Terminase, Head, Tail |  |  |
| F440-C | 7 | Incomplete | NA | 1 | Toxin- B |
|  | 11.2 | Incomplete | Protease, Tail |  |  |
| Z197-ii | 26 | Incomplete | Integrase | 1 | EFV |
|  | 19.8 | Questionable | Tail, Terminase, Portal, Head |  |  |
| F793 | 5.9 | Incomplete | Transposase, Tail | 6 | Collagen adhesin |
| AN540 | 6.9 | Questionable | Transposase, Integrase | 3 | CFA/I fimbrial subunit D,  Multidrug export protein (MepA),  SrrA |
| AN570 | 7 | Incomplete | NA | 3 | Internalin-J, Nucleoid occlusion protein |
| 294-AKM3-iv | 7 | Incomplete | NA | 3 | Internalin-J, Nucleoid occlusion protein |
|  | 28.5 | Incomplete | Transposase, Integrase |  |  |
| VI11-2Lu | 25.3 | Incomplete | Tail, Head, Terminase | 2 | Toxin B, EFV, Nucleoid occlusion protein |
|  | 16.7 | Incomplete | NA |  |  |
|  | 11.2 | Incomplete | Transposase |  |  |
| VI10-23 | 36.6 | Questionable | Integrase, Head, Tail | 2 | Toxin B, EFV, Nucleoid occlusion protein |
| SKnolD1 | 23.4 | Incomplete | Transposase, Recombinase | 4 | Internalin-J, Nucleoid occlusion protein |
| 6028 | 39.6 | Incomplete | Head, Tail | 8 | Tetracycline resistance protein (TetM) from transposon TnFO1,  SrrA, Collagen adhesin, Bleomycin resistance protein, Streptomycin 3’-adenylyltransferase, Kanamycin nucleotidyltransferase |
|  | 19.5 | Incomplete | Tail, Transposase, Integrase |  |  |
| 8929 | 18.9 | Incomplete | Integrase, Transposase, Tail | 6 | TetM, EFV, Collagen adhesin |
|  | 27.3 | Incomplete | Integrase |  |  |
|  | 18.4 | Incomplete | Terminase, Portal, Head, Tail |  |  |
| B2411 | 19.8 | Incomplete | Terminase, Head, Tail | 4 | Collagen adhesin, EFV |
| B1037 | 9 | Incomplete | Integrase, Capsid | 5 | Collagen adhesin, EFV |
|  | 18 | Incomplete | Terminase, Head, Tail |  |  |
| B3129 | 27.2 | Intact | Transposase, Tail, Capsid, Protease, Portal, Terminase | 7 | Aminoglycoside 6-adenylyltransferase, Streptomycin 3''-adenylyltransferase, Collagen adhesin, TetM |
|  | 39.3 | Incomplete | Tail |  |  |
|  | 25.3 | Incomplete | Integrase |  |  |
|  | 5.9 | Incomplete | Tail, Transposase |  |  |
| MX10-3PSLN | 36.6 | Questionable | Integrase, Head, Tail | 3 | - |
| 600-16626-4bBM | 22.5 | Incomplete | Tail, Head, Terminase | 6 | Internalin- J, EFV, Nucleotide occlusion protein |
| 600-1626-5BM | 18.6 | Incomplete | Terminase, Head, Tail | 4 | Internalin- J, EFV, Nucleotide occlusion protein |
| 600-16262-14BM | 7 | Incomplete | NA | 6 | Internalin- J, EFV, Nucleotide occlusion protein |
| Banks173-Banks1 | 25.3 | Incomplete | Tail, Head, Terminase | 2 | EFV, Nucleotide occlusion protein |
|  | 16.7 | Incomplete | NA |  |  |
|  | 11.2 | Incomplete | Transposase |  |  |
| Banks174-K | 25.3 | Incomplete | Tail, Head, Terminase | 2 | EFV, Nucleotide occlusion protein |
|  | 16.7 | Incomplete | NA |  |  |
|  | 11.2 | Incomplete | Transposase |  |  |
| Banks971-Li | 25.3 | Incomplete | Tail, Head, Terminase | 2 | EFV, Nucleotide occlusion protein |
|  | 16.7 | Incomplete | NA |  |  |
|  | 12.3 | Incomplete | Transposase |  |  |
| D170.2-Sp | 20 | Incomplete | Terminase, Head, Tail | 2 |  |
|  | 11.2 | Incomplete | Transposase |  |  |
| D969-Sp | 17.8 | Incomplete | NA | 2 | Toxin-B, Nucleotide occlusion protein |
|  | 19.6 | Incomplete | Terminase, Head, Tail |  |  |
|  | 11.2 | Incomplete | Transposase |  |  |
| D971-Sp | 25.3 | Incomplete | Tail ,Head, Terminase | 2 | EFV, Nucleotide occlusion protein |
|  | 16.7 | Incomplete | NA |  |  |
|  | 25.3 | Incomplete | Transposase, Integrase |  |  |
| D972-Sp | 25.3 | Incomplete | Tail, Head, Terminase | 2 | EFV, Nucleotide occlusion protein |
|  | 16.7 | Incomplete | NA |  |  |
|  | 25.6 | Incomplete | Transposase, Integrase |  |  |
| MX10-1fe | 25.3 | Incomplete | Tail, Head ,Terminase, Integrase | 2 | Toxin-B, EFV, Nucleotide occlusion protein |
|  | 16.7 | Incomplete | NA |  |  |
|  | 11.2 | Incomplete | Transposase |  |  |
| MX10-2Lu | 25.3 | Incomplete | Tail, Head, Terminase | 2 | Toxin-B, EFV, Nucleotide occlusion protein |
|  | 16.7 | Incomplete | NA |  |  |
|  | 11.2 | Incomplete | Transposase |  |  |
| MX11-1fe | 8.7 | Incomplete | Transposase | 2 | Toxin-B, EFV, Nucleotide occlusion protein |
|  | 30.4 | Incomplete | NA |  |  |
|  | 21.4 | Incomplete | Terminase, Head, Tail |  |  |
|  | 11.2 | Incomplete | Transposase |  |  |
| MX11-2MLN | 30.4 | Incomplete | NA | 2 | Toxin-B |
|  | 21.4 | Incomplete | Terminase, Head, Tail |  |  |
|  | 11.2 | Incomplete | Transposase |  |  |
| MX3-Lu | 30.7 | Incomplete | NA | 2 | Toxin-B, EFV, Nucleotide occlusion protein |
|  | 21.4 | Incomplete | Terminase, Head, Tail |  |  |
|  | 11.2 | Incomplete | Transposase |  |  |
| 593-EU10-BM | 25.3 | Incomplete | Tail, Head, Terminase | 2 | Toxin-B, EFV, Nucleotide occlusion protein |
|  | 16.7 | Incomplete | NA |  |  |
|  | 11.2 | Incomplete | Transposase |  |  |
| 593-EU08-BM | 30.4 | Incomplete | NA | 2 | Toxin-B, EFV, Nucleotide occlusion protein |
|  | 21.4 | Incomplete | Terminase, Head, Tail |  |  |
|  | 11.2 | Incomplete | Transposase |  |  |
| 593-EU05-BM | 25.3 | Incomplete | Tail, Head, Terminase | 3 | EFV, Nucleotide occlusion protein |
|  | 16.7 | Incomplete | NA |  |  |
|  | 11.2 | Incomplete | Transposase |  |  |
| 593-EU04-K | 25.3 | Incomplete | Tail, Head, Terminase | 3 | EFV, Nucleotide occlusion protein |
|  | 16.7 | Incomplete | NA |  |  |
|  | 11.2 | Incomplete | Transposase |  |  |
| 593-EU03-BM | 25.3 | Incomplete | Tail, Head, Terminase | 2 | EFV, Nucleotide occlusion protein |
|  | 16.7 | Incomplete | NA |  |  |
|  | 11.2 | Incomplete | Transposase |  |  |
| 593-EU02-S | 25.3 | Incomplete | Tail, Head, Terminase | 3 | EFV, Nucleotide occlusion protein |
|  | 16.7 | Incomplete | NA |  |  |
|  | 11.2 | Incomplete | Transposase |  |  |
| 593-EU01-S | 25.3 | Incomplete | Tail, Head, Terminase | 2 | Toxin-B, EFV, Nucleotide occlusion protein |
|  | 16.7 | Incomplete | NA |  |  |
|  | 11.2 | Incomplete | Transposase |  |  |
| 594-1BM | 22.8 | Incomplete | Integrase | 4 | CFA/I fimbrial subunit D, MepA, EFV |
|  | 20.6 | Incomplete | Terminase, Head, Tail |  |  |
|  | 11.2 | Incomplete | Transposase |  |  |
| 364-ER25-H | 17.9 | Incomplete | Head, Tail | 2 | EFV, Nucleoid occlusion protein |
| 459-ER29-In | 19.6 | Incomplete | Terminase, Head, Tail | 2 | EFV, Nucleoid occlusion protein |
| 459-ER31-Lu | 23.6 | Incomplete | Tail, Head, Terminase | 2 | EFV |
| 459-ER33-BM | 19.6 | Incomplete | Terminase, Head, Tail | 2 | EFV, Nucleoid occlusion protein |
| 459-ER34-BM | 10 | Incomplete | Tail | 2 | Toxin-B, EFV |
|  | 11.2 | Incomplete | Protease, Tail |  |  |
|  | 8.9 | Incomplete | NA |  |  |
|  | 7.8 | Incomplete | Head |  |  |
| 459-ER41-BM | 18 | Incomplete | Tail, Head, Terminase | 2 | EFV |
| 459-ER42-SI | 18 | Incomplete | Terminase, Head, Tail | 2 | EFV |


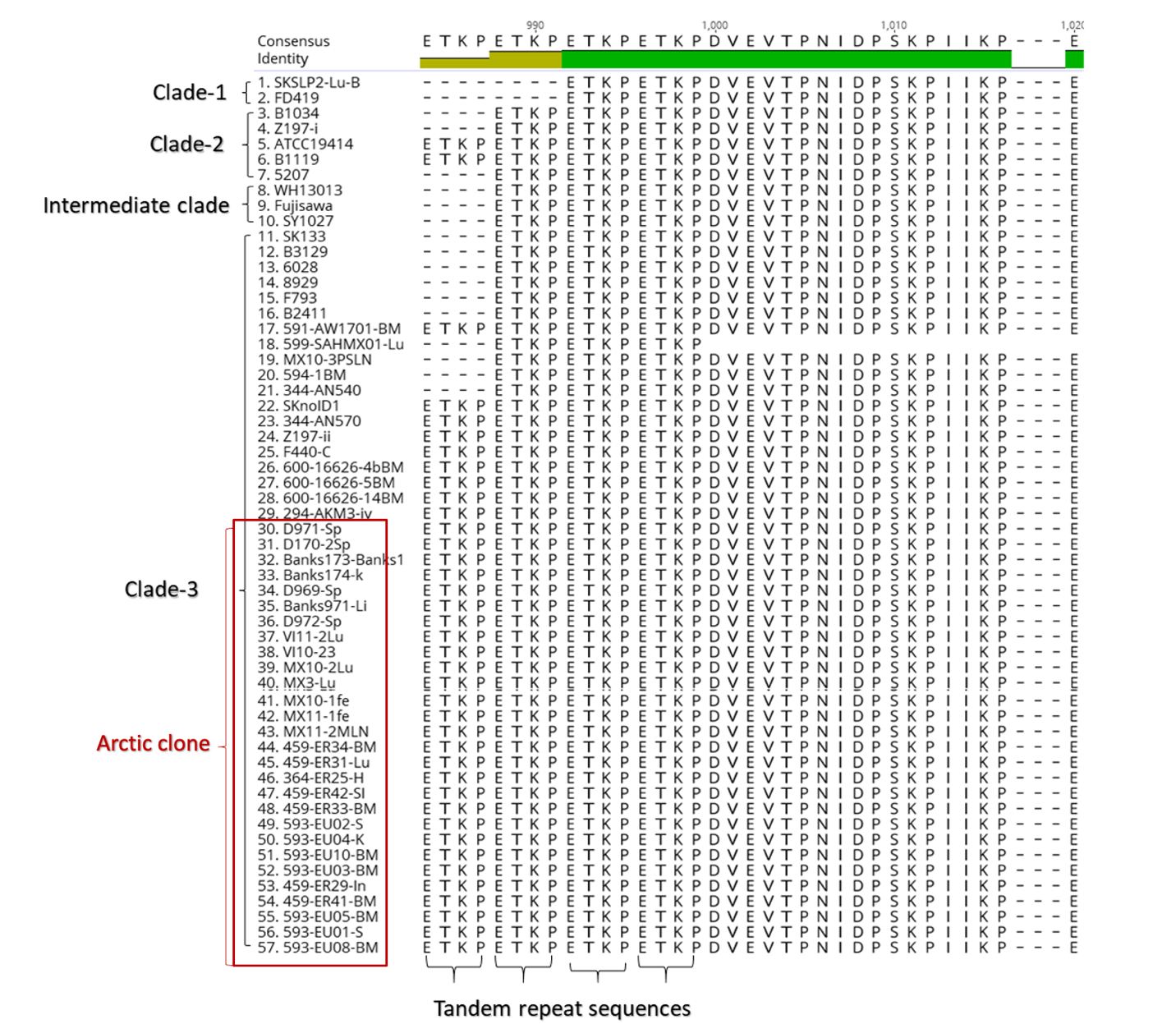


**Figure S1. Multiple sequence alignment of hyaluronidase amino acid sequence from 57 *E. rhusiopathiae* genomes.** Variation in the number of tandem repeat sequences (ETKP) can be observed.

**Supplementary References**

1. Yang L, Zhu Y, Peng Z, Ding Y, Jie K, Wang Z, et al. Comparative genome analysis of a pathogenic Erysipelothrix rhusiopathiae isolate WH13013 from pig reveals potential genes involve in bacterial adaptions and pathogenesis. Veterinary Sciences. 2020;7:74.

2. Ogawa Y, Ooka T, Shi F, Ogura Y, Nakayama K, Hayashi T, et al. The genome of Erysipelothrix rhusiopathiae, the causative agent of swine erysipelas, reveals new Insights into the evolution of Firmicutes and the organism’s intracellular adaptations. Journal of Bacteriology. 2011;193:2959–71.

3. Kwok AH, Li Y, Jiang J, Jiang P, Leung FC. Complete genome assembly and characterization of an outbreak strain of the causative agent of swine erysipelas – Erysipelothrix rhusiopathiae SY1027. BMC Microbiology. 2014;14:176.

4. Forde TL, Orsel K, Zadoks RN, Biek R, Adams LG, Checkley SL, et al. Bacterial genomics reveal the complex epidemiology of an emerging pathogen in Arctic and boreal ungulates. Frontiers in Microbiology. 2016;7.

5. Mavrot Fabien, Forde Taya, Tomaselli Matilde, Anderson Morgan, Kutz Susan. Studying rare occurrences: long-term phylogenetic monitoring of the emerging pathogen Erysipelothrix rhusiopathiae during wildlife mortality events. Online: ArcticNet; 2020.
